# Discovery of relict subglacial lakes and their geometry and mechanism of drainage

**DOI:** 10.1038/ncomms11767

**Published:** 2016-06-13

**Authors:** Stephen J. Livingstone, Daniel J. Utting, Alastair Ruffell, Chris D. Clark, Steven Pawley, Nigel Atkinson, Andrew C. Fowler

**Affiliations:** 1 Department of Geography, Sheffield University, Winter Street, Sheffield S10 2TN, UK; 2 Alberta Geological Survey, Edmonton, Alberta T6B 2X3, Canada; 3 School of Geography, Archaeology and Palaeoecology, Queen's University Belfast, Northern Ireland BT9 6AY, UK; 4 Mathematics Applications Consortium for Science and Industry (MACSI), University of Limerick, Limerick, Ireland; 5 Mathematical Institute, University of Oxford, Oxford OX2 6GG, UK

## Abstract

Recent proxy measurements reveal that subglacial lakes beneath modern ice sheets periodically store and release large volumes of water, providing an important but poorly understood influence on contemporary ice dynamics and mass balance. This is because direct observations of how lake drainage initiates and proceeds are lacking. Here we present physical evidence of the mechanism and geometry of lake drainage from the discovery of relict subglacial lakes formed during the last glaciation in Canada. These palaeo-subglacial lakes comprised shallow (<10 m) lenses of water perched behind ridges orientated transverse to ice flow. We show that lakes periodically drained through channels incised into bed substrate (canals). Canals sometimes trend into eskers that represent the depositional imprint of the last high-magnitude lake outburst. The subglacial lakes and channels are preserved on top of glacial lineations, indicating long-term re-organization of the subglacial drainage system and coupling to ice flow.

## Abstract (web summary)

Our understanding of subglacial lake drainage events is limited by a lack of direct observations. Here, the authors use ground penetrating radar to identify a relict subglacial lake and infer drainage mechanisms based on geomorphological features.

## Graphical abstract

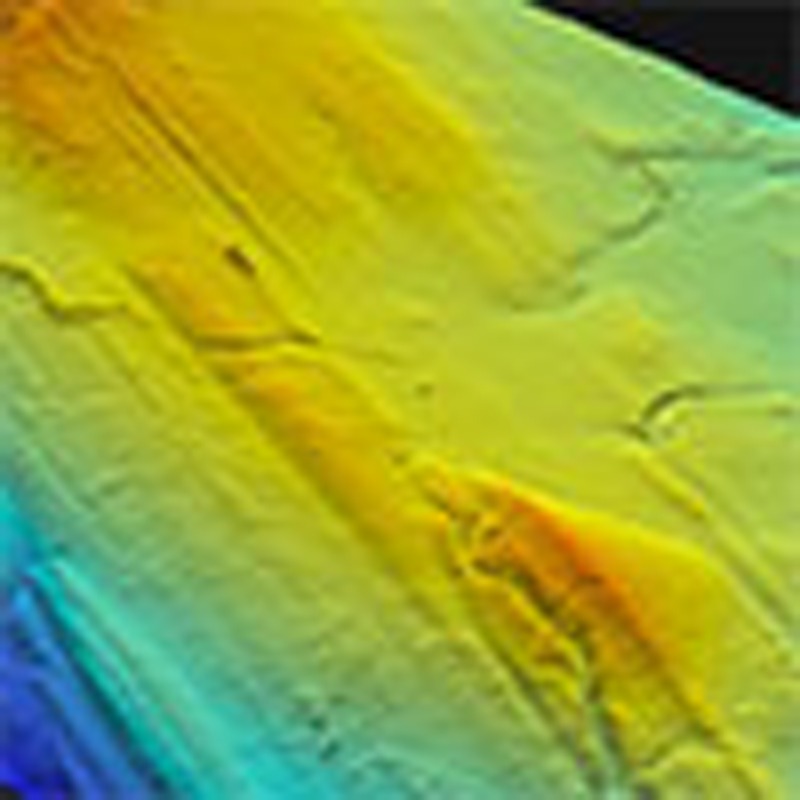

There are hundreds of reported and thousands of predicted subglacial lakes beneath the Antarctic and Greenland ice sheets^1^,^2^. Subglacial lakes provide a unique microbial ecosystem and depositional environment capable of providing insight into past ice sheet evolution and stability^3^,^4^. Lake drainage is documented by deflation of the overlying ice surface on annual timescales^5^,^6^,^7^. Drainage is thought to occur over hundreds of kilometres through the subglacial hydrological network and may involve discharge into downstream lakes. However, we have insufficient knowledge of the lake-drainage process (that is, how drainage initiates and proceeds) to properly constrain its dynamic impact^8^,^9^,^10^. Postulated channelized drainage of subglacial lakes^5^,^11^ is based on observations and modelling of alpine jökulhlaup systems, where the formation of conduits incised into the ice (Röthlisberger-channels) is well established^12^,^13^. However, the longer duration drainage of lakes beneath ice sheets implies a different subglacial hydraulic regime^14^ and is a key unknown. Do lakes drain through channelized conduits or distributed sheets/cavities, and how do they evolve downstream? If drainage is via conduits, how many are there, are they incised into sediment or ice, and what is their geometry?

Palaeo-subglacial lakes are widely predicted beneath the former Laurentide Ice Sheet^11^,^15^, but investigation of their geological signature and drainage pathways are rare^16^,^17^,^18^ because of the difficulty of distinguishing between proglacial and subglacial lake sediments^19^,^20^. In this paper, based on a range of sedimentary, geomorphological and geophysical techniques, we identify relict subglacial lakes and their drainage routeways in Alberta, Canada. The study area encompasses the proposed position of an ice saddle resulting from the coalescence of the Laurentide and Cordilleran ice sheets where model predictions indicate a proclivity for subglacial meltwater convergence and ponding^15^. Our findings suggest subglacial lakes comprised shallow lenses of water perched behind ridges orientated transverse to ice flow. Lake drainage occurred repeatedly through channel(s) incised into bed substrate, some of which evolve downstream into R-channels cut up into the overlying ice. The formation of subglacial lakes beneath this portion of the Laurentide Ice Sheet may have been associated with the cessation of fast ice-flow.

## Results

### Geomorphology

**Figure 1:**
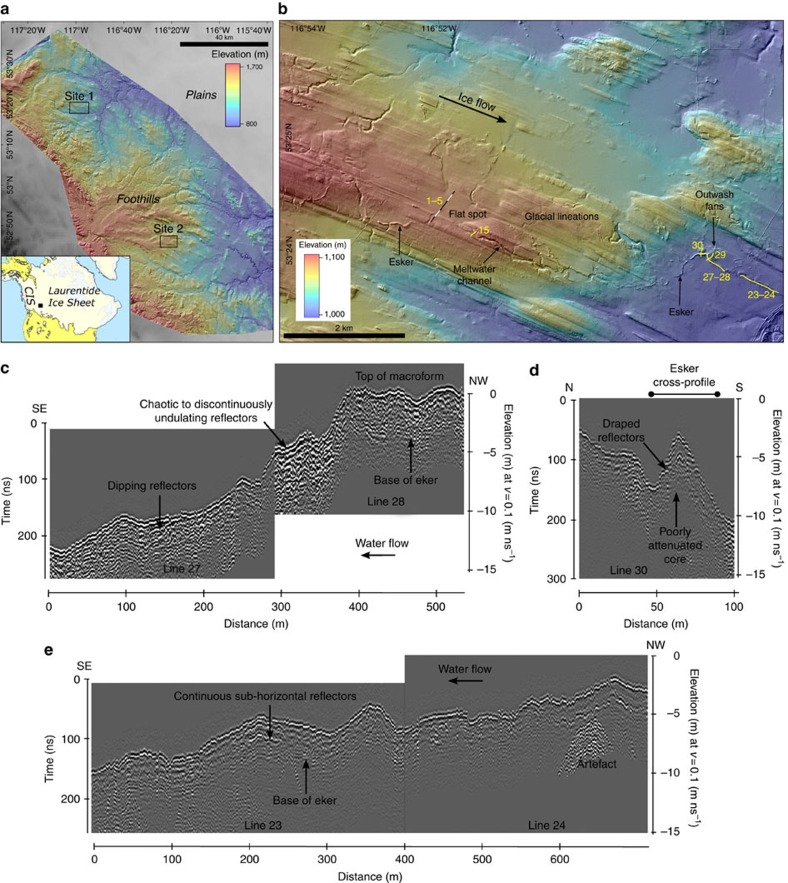
Glacial geomorphology and GPR profiles at Site 1. (**a**) Location of the palaeo-subglacial lakes in Alberta, Canada. The coloured elevation gradient is the extent of the LiDAR data from which we conducted the mapping. Site 1 is shown in **b** and Site 2 is shown in Fig. 2a. CIS=Cordilleran Ice Sheet. (**b**) Glacial geomorphology of Site 1 showing the location of a palaeo-subglacial lake (flat spot) connected by a number of inflowing and outflowing subglacial meltwater channels. Note the downstream transition of the main subglacial meltwater channel into an esker. Lines 1–5 are a GPR profile across the flat spot (Supplementary Fig. 5). Line 15 is a GPR profile across a meltwater channel (Supplementary Fig. 4). Lines 23–34, 27–28 and 30 are GPR lines shown in **c**–**e**. See Supplementary Fig. 1a for mapping of flat spots, meltwater channels, eskers and outwash fans. (**c**) GPR long profile of esker running downstream from the top of the macroform. (**d**) GPR cross-profile of esker showing reflectors that drape a poorly attenuated core. (**e**) GPR long profile of esker downstream of the macroform.

**Figure 2:**
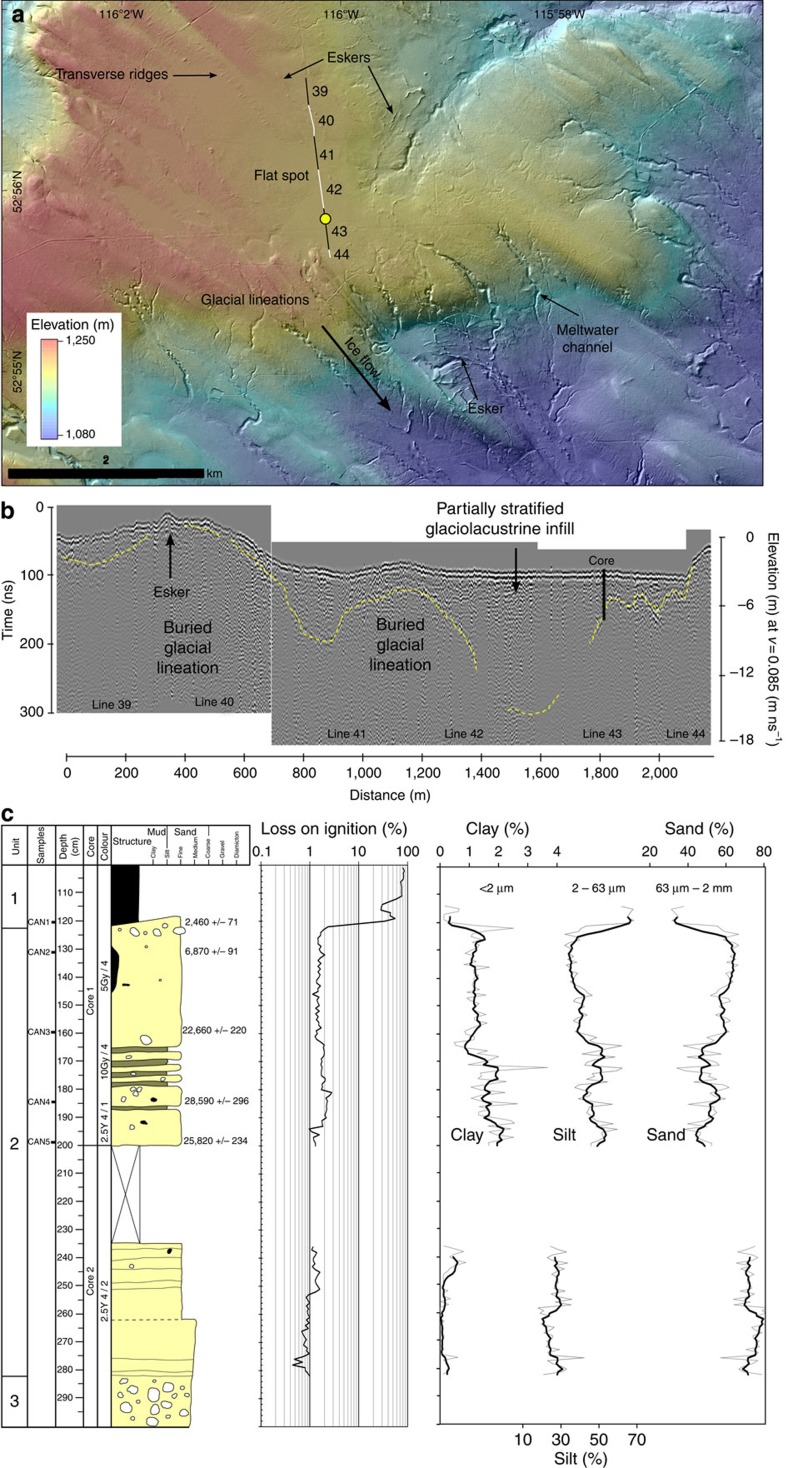
Glacial geomorphology at Site 2 and its geophysical and sedimentological characteristics. (**a**) Glacial geomorphology of Site 2, showing the location of a palaeo-subglacial lake (flat spot) connected to a number of inflowing and outflowing subglacial meltwater channels and eskers. Lines 39–44 are a GPR profile shown in **b**. Yellow dot is the location of sediment core in **c**. See Supplementary Fig. 1b for mapping of flat spots, meltwater channels, eskers, crevasse squeeze ridges and outwash fans. (**b**) GPR profile across the flat spot showing the burial of glacial lineations in up to 10 m of glaciolacustrine sediments (Supplementary Fig. 6 shows the raw data). (**c**) Sediment log of a core recovered near the edge of a flat spot. This includes calibrated radiocarbon ages displayed with 2 sigma error, LOI data and particle size characteristics (see the Methods—Sedimentology for details).

**Figure 3:**
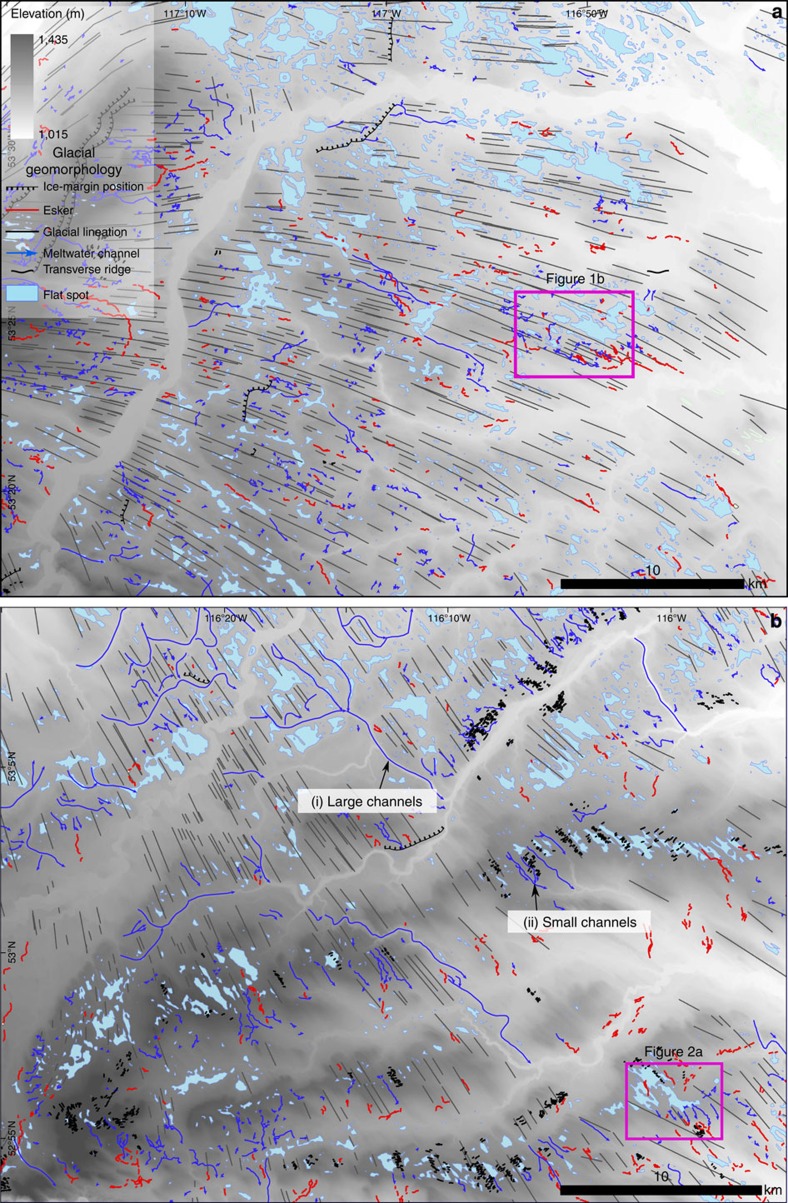
Glacial geomorphological mapping. See the methods—Geomorphology for details. (**a**) Site 1 (Fig. 1b) and surrounding region, (**b**) Site 2 (Fig. 2a) and surrounding region. Mapping reveals a large number of flat spots that represent potential palaeo-subglacial lakes. They typically occur in linear clusters perched behind upland ridges running transverse to ice flow. There are two scales of drainage system (marked on **b**): (i) large (subglacial/proglacial) meltwater spillways that are not connected with flat spots, which cut across the transverse ridges, and (ii) small subglacial channels and associated eskers that often lead into and out of flat spots.

High-resolution mapping of glacial bedforms from LiDAR in the foothills of central Alberta, western Canada (see the Methods for details), reveal linear clusters of <5 km diameter flat spots connected by subglacial meltwater channels and eskers (Figs 1, 2). These flat spots tend to occur behind low-relief (<10 m) geologically controlled ridges and palaeo-valleys aligned perpendicular to south east (SE) orientated glacial lineations (Fig. 3). We documented 51 locations within the 11,000 km^2^ study area where channels emanate from flat spots of which 11 connect to another flat spot downstream. In eight localities, meltwater channels show a downstream transition into 10–40 m wide by 1–10 m high eskers (for example, Fig. 1b). The channels are either aligned roughly parallel to the former ice-flow direction or down local slopes (Figs 1b and 2a and Supplementary Fig. 1). Channels that are connected to palaeo-subglacial lakes have undulating thalwegs that cut across downstream ridges and are typically 200–300 m wide, 4–10 m deep and between 700 and 1,700 m long. The cross-sectional area of the channels is not related to the size of the flat spots (Supplementary Fig. 2).

### Sedimentology and stratigraphy

We acquired ground-penetrating radar (GPR) across two flat spots and a sediment core (see the Methods for details) at the edge of the flat spot at Site 2. The coring site was chosen where we could penetrate through the lacustrine sediments into the underlying glacial lineation, based on GPR investigation of thick sediment infills. The results revealed that the flat spots are composed of 5- to 10-m-thick glaciolacustrine sediments overlying glacial lineations (Fig. 2b,c). The 3-m sediment core (Fig. 2c) contained an upper 1.2 m unit of herbaceous peat (unit 1), a middle 1.6 m unit comprising horizontally laminated silt and fine sand, and diffusively laminated to massive sandy-silt and silty-sand with rare outsized gravel and pebbles (unit 2), and a lower 0.2 m unit of silty-sand with frequent gravel and pebbles that may penetrate into a glacial lineation (unit 3). The organic carbon content of the sediments ranges from 40–86% for unit 1 to 0.4–2.8% in unit 2. A sample taken at the base of the herbaceous peat unit at 120–121 cm depth provided an age of 2,460±71 cal. before present (BP). Four bulk samples were taken from the middle of unit 2 (130–199 cm depth) and sampled from the fine sediment fraction at intervals with relatively high organic carbon content. In down-core order, these provided ages of 6,870±91 cal. BP, 22,660±220 cal. BP, 28,590±296 cal. BP and 25,820±234 cal. BP, respectively.

At Site 1, GPR data were collected across a round-crested esker deposited downstream of a meltwater channel (Fig. 1c–e). The esker varies in size, has an undulating morphology and exhibits a sharp change in direction about halfway along its length that coincides with esker enlargement and the deposition of an outwash fan. GPR cross-profiles reveal a central poorly attenuated core draped by reflectors that can be traced across the ∼20 m width of the esker (Fig. 1d). In long profile, reflectors can vary from chaotic to discontinuously undulating and downstream dipping up-esker (Fig. 1c), to continuous sub-horizontal reflectors further down-flow (Fig. 1e).

## Discussion

In summary, mapping of glacial landforms from high-resolution digital elevation models (DEMs) revealed the presence of flat spots associated with channels and eskers (Figs 1, 2, 3). Investigation of the structure and sediments of two flat spots suggests they comprise <10 m of glaciolacustrine sediment (Fig. 2b,c). A subglacial lake origin is favoured because of their association with subglacial meltwater channels and eskers (Figs 1b and 2a), some of which appear to cut into the glaciolacustrine sediments and must therefore have formed after the flat spot. In contrast, a proglacial lake origin is difficult to reconcile with the open configuration of many of the flat-spots (Figs 1b and 2a), requiring awkward ice-margin positions to dam the basins and pond water during north west (NW) ice retreat (Supplementary Fig. 3). Moreover, the deposition of thick basin infills (up to ∼10 m) is at odds with well-preserved subglacial bedforms not overprinted by moraines or thick deglacial deposits, marking still-stand positions, plus there is no evidence of upwards fining indicative of a retreating ice margin. Our discovery of palaeo-subglacial lakes and their drainage architecture therefore provides a unique opportunity to characterize its depositional archive and drainage imprint, and to assess the latest theories for lake drainage.

Given the geometry of the flat spots (hundreds of metres to few kilometres in diameter), the thickness of the sediment fill (<∼10 m) and the subdued relief of the glacially lineated topography, the lakes must have existed as shallow (few m deep), sediment-floored water lenses. Modelling of the hydraulic potential surface^15^ suggests low ice-surface slopes (1–2 m/km) are required to form the lakes. This is analogous to many of the ‘active' subglacial lakes found beneath low-gradient ice streams in Antarctica^21^. The spatial distribution of lakes perched behind ridges orientated transverse to ice flow and spaced at ∼5–10 km intervals is comparable in scale and arrangement to low basal shear stress regions (that is, slippy patches) between rib-like patterns of high basal shear stress (that is, sticky patches), revealed by inversions of basal shear stress beneath the Antarctic Ice Sheet^22^. However, in our case, variations in basal friction would have arisen from the bed topography (that is, water damming behind ridges) rather than dynamic instabilities in the effective pressure^22^.

**Figure 4:**
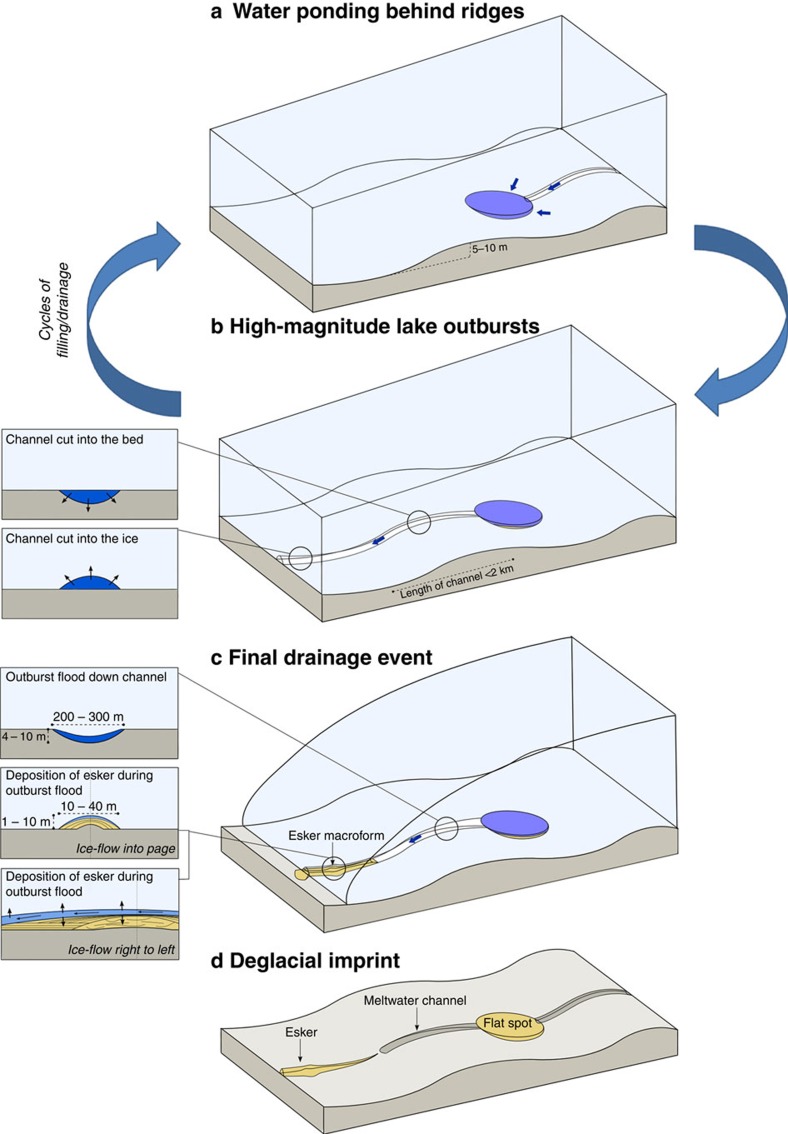
Conceptual model and geometries of periodic subglacial lake drainage. (**a**) Water ponding behind transverse ridges is shown; when the lake fills or the water pressure becomes high enough water drains across the ridge (**b**). This occurs through a canal cut down into the sediment that evolves downstream into an R-channel cut up into the ice (**b**). Filling and draining of the lake may occur many times, with the final drainage depositing an esker in the R-channel (**c**). (**d**) The relict lake and its drainage imprint are shown. Graphic is not to scale.

The drainage of subglacial lakes is typically assumed to occur through Röthlisberger-channels incised in ice (R-channels)^5^,^11^. However, we demonstrate the potential for periodic drainage through channels eroded into the substrate (canals; Fig. 4 and Supplementary Fig. 4). This is consistent with more recent modelling studies, which require greater rates of tunnel closure by till deformation to produce the long-duration drainage events observed beneath Antarctica^15^. The channels were probably incised during a series of basal meltwater production, storage and drainage cycles as their cross-sectional area is not related to the size of the palaeo-subglacial lakes (Supplementary Fig. 2). The downstream transition from meltwater channels (canals) into 10–40 m wide by 1–10 m high eskers (for example, Fig. 1b), demonstrates both an evolution from canals to R-channels, and a switch from sediment erosion to deposition (Fig. 4c). Deposition is typically associated with a drop in water velocity, which will occur when there is a reduction in the hydraulic gradient. This may arise from a change in the slope of the bed or ice, and/or a change in effective pressure, and is consistent with the formation of eskers at the toe of bed slopes, and near the ice margin as evidenced by outwash fans. Indeed, the formation of an esker-fan 4 km downstream of the palaeo-subglacial lake at Site 1 (Fig. 1b) reveals the potential for subglacial lakes to persist beneath thin ice close to the glacier margin. An implication of water drainage through R-channels is that they may not leave a geomorphic imprint if sediment supply is limited. The switch from canals to R-channels is consistent with our current theoretical understanding of subglacial floods (see Supplementary Discussion and Supplementary Figs 7 and 8 for details)^14^,^23^. The form of a channel (that is, whether a canal or R-channel develops) is strongly dependent on the effective pressure, *N* (ice overburden pressure—water pressure). An increase in *N* will cause ice viscosity to decrease and sediment stiffness to increase such that canals will tend to form when *N* is high, whereas R-channels are favoured when *N* is low. Critically, the flood theory predicts a decrease in *N* over a fairly short distance adjacent to the lake and the length of this region is about a kilometre, which is roughly consistent with the observed length scale of the meltwater channels (<2 km). Thus, the observed transition from canals to R-channels is consistent with theory and may occur if the ratio of sediment stiffness to ice viscosity passes through one.

GPR investigation of an esker downstream of a subglacial lake and meltwater channel (Fig. 1c–e) has allowed the identification of a number of distinctive facies. The central, poorly attenuated core, which is typical of fine-grained sediment such as diamicton or bedrock (Fig. 1d), may have been squeezed into the low-pressure conduit during a period of low flow and sediment supply^24^,^25^. Reflectors draped over the core can be traced across the ∼20-m width of the esker, indicating water flow across the entire width of the esker. Chaotic and discontinuous reflectors are consistent with rapid anti-dune aggradation of coarse bedload in low-flow depths (<3 m) and high-flow velocities^25^. Downstream dipping reflectors towards the top of the sequence relate to foreset accretion in the lee of a depocentre. This macroform manifests as a ridge enlargement at the surface and suggests conduit enlargement during high sediment supply and flow velocity^25^,^26^. Downstream of the esker macroform, continuous sub-horizontal reflectors record vertical accretion by a high-velocity flow where tunnel growth exceeds sediment supply creating accommodation space^25^. Together, the GPR data across the esker are consistent with observations of high-magnitude outburst flood eskers formed during a single, rapid drainage event^26^. The esker, therefore, represents the final drainage of the palaeo-subglacial lake along an at least 10–40 m wide by 1–10 m high R-channel (Fig. 4e). If most of the material in the esker (volume: ∼130,000 m^3^) was derived from the upstream canal (volume: ∼560,000 m^3^), then at least four similar-sized lake drainage events are required to erode the composite canal. Thus, we interpret this as evidence for periodic jökulhlaup-style floods beneath contemporary ice sheets, capable of eroding and depositing large volumes of subglacial sediment. This is consistent with work in NE Alberta that invoke periodic high-magnitude outbursts from subglacial lakes to explain the evolution of large (up to several kilometres wide, tens of kilometres in length and hundreds of metres deep) tunnel valleys cut into sediment^27^.

The depositional environment of the middle unit (2) in the sediment core is interpreted as a low-energy ice-proximal water body dominated by fine-grained outwash and rain-out sedimentation, which is consistent with a subglacial lake origin. The organic carbon content of the lake sediments (0.4–2.8%) is within the range of sediments sampled from small subglacial lakes emerging from beneath the Antarctic Ice Sheet^3^. Preservation of horizontal laminations and the absence of a capping till or features associated with shearing suggest that overriding ice did not recouple with the bed and erode sediments from the lake^3^. Thus, the lake must have emerged from under the ice during deglaciation at ∼16 ka BP or the ice exerted low basal shear stress, likely due to stagnation. This hypothesis is supported by a mid-Holocene age of 6,870±91 cal. BP towards the top of unit 2, which suggests a transition to a subaerial lake environment, with the initiation of peat formation occurring during the late Holocene at 2,460±71 cal. BP. The lowest three ages range from 23 to 29 cal. ka BP and are not in stratigraphic order, which is consistent with a subglacial lake origin, whereby much of the carbon was derived from allochthonous older soils that pre-date the last phase of glacial activity (∼26–16 ka BP)^28^ and from surface and basal melt inputs.

Development of palaeo-subglacial lakes and the preservation of their associated deposits above glacial lineations (Figs 1b, 2a and 3) demonstrate a significant hydrological reorganization of the ice-sheet bed during the last deglaciation. The glacial lineations are the imprint of a SE-flowing terrestrial ice stream^29^, which is typically thought to be strongly influenced by the presence of saturated basal sediments and abundant distributed meltwater, facilitating enhanced deformation and/or sliding of ice across its bed^30^. We interpret the switch from glacial lineations to subglacial lakes as a record of shut-down of ice streaming, initiated by or resulting in, the development of a channelized drainage system. Critically, fast ice flow will lower the ice-surface slope, which is one means of increasing the potential for subglacial ponding^6^ and triggering a switch in meltwater regime to one dominated by channelized drainage of subglacial lakes. The long-term development of such an efficient and localized water storage and drainage network capable of withdrawing water from other areas of the bed may be to increase basal resistance, causing ice-stream deceleration or even stagnation, as suggested for the Whillans Ice Stream, West Antarctica^31^. Thus, although lake drainage events may cause transient increases in ice flow^5^, we also propose the importance of long-term hydro-dynamic feedbacks, which might do the opposite.

To our knowledge, this is the first time former subglacial lakes and their drainage architecture have been directly observed beneath the North American ice sheets, and therefore provides a unique glimpse into one of the least explored and understood environments on Earth. We are able to provide new constraints on the mechanism and geometry of subglacial lake drainage that are consistent with our current theoretical understanding. We also show the potential for long-term hydro-dynamic re-organizations, demonstrated by the association of subglacial lakes with the cessation of fast ice-flow in the region. Our results provide constraints for the modelling of similar subglacial lake drainages beneath the Antarctic and Greenland ice sheets.

## Methods

### Ground-penetrating radar

A MALA Ramac system was deployed, comprising a 100-MHz rough terrain antenna array (RTA), linked by fibre optic cables to a backpack communication unit and thence an XV11 control monitor. The RTA is a 4-m-long cable with transmitting and receiving antennas in an in-line endfire orientation. This antenna is ideal for the extremely rugged environments surveyed. Lines of roughly 1 km length were located and heights above ordnance datum (OD) recorded using a Garmin E30 GLONASS and global positioning system (GPS) enabled hand-held Global Navigation Satellite System device, with 2–4 m lateral accuracy. A total of 45 km line length was gathered, of which we show 2.7 km here. The remaining data are available as images upon request to the first author. Data were gathered in continual trigger mode, with 16 stacks at a 0.5-s delay at a constant walking speed, checked by Global Navigation Satellite System positions. Data were processed using Sandmeier's proprietary software ReflexW (Queen's University Licence number 401). This comprised de-wow, trace averaging, application of gain, migration, topographic correction and depth conversion. A limitation of the RTA is these antennas are inseparable, which precludes wide-angle reflection experiments to determine subsurface velocities. Instead, the hyperbola-fitting function within ReflexW was used to check the ground-truthed sediment type and depth control afforded by our 3-m-deep core. A velocity of 0.085 m ns^−1^ was calculated across the flat spots, in accordance with silt and sand dominated sediments, and a velocity of 0.1 m ns^−1^ was calculated for the eskers, which is in accordance with other GPR surveys of eskers in Alberta^25^.

### Sedimentology

A surface sediment core was collected using a percussion hammer corer to a total sediment depth of 3 m. Some sediment was extruded from the core upon bringing to the surface leading to a gap in the data from 200 to 235 cm. Physical characteristics were manually described and logged. The core was subsampled every 1 cm to determine organic and carbonate content based on % weight loss on ignition after combustion at 430 °C for 24 h. Particle sizes of the <2 mm fraction (sand–silt–clay) were determined using a Horiba laser defraction system. Samples were dated from the base of the herbaceous peat unit (120–121 cm) together with four bulk samples from relatively ‘organic-rich' sections of the core (Fig. 2c). The four bulk samples were selected because macrofossils were absent beneath the peat unit. Accelerator mass spectrometry (AMS) radiocarbon dating was carried out at the Queen's University (Belfast) CHRONO^14^ Radiocarbon Laboratory. Radiocarbon ages were calibrated with the Calib 6.0.2 program using IntCal13 (ref. 32).

### Geomorphology

LiDAR data were collected and processed by Airborne Imaging and are used under licence from Clean Harbors Exploration Services Ltd. The data were collected from a fixed wing aircraft between 23 May and 20 August 2006, using an Optech ALTM 3100. Data were classified using the programme Terrascan to generate a bare earth digital elevation model, which has an horizontal accuracy 45 cm root mean square (RMS) and a vertical accuracy 30 cm RMS. We manually digitized glacial lineations, transverse ridges (moraines), eskers and meltwater channels from 2 m LiDAR data. Flat spots were calculated using the standard deviation of the elevation in a 100-m window. By calibrating to known palaeo-subglacial lakes in Figs 1 and 2a, we used a cutoff standard deviation of <0.6 m to pick out flat spots. Flat spots associated with modern fluvial systems and former proglacial lakes were identified and omitted.

The data that support the findings of this study are available from the corresponding author upon request.

## Additional information

**How to cite this article:** Livingstone, S. J. *et al.* Discovery of relict subglacial lakes and their geometry and mechanism of drainage. *Nat. Commun.* 7:11767 doi: 10.1038/ncomms11767 (2016).

## Supplementary Material

Supplementary Information Supplementary Figures 1-8, Supplementary Discussion and Supplementary References
