# Supplementary material for: Discovery of relict subglacial lakes and their geometry and mechanism of drainage
Source: Nat Commun. 2016 Jun 13;7:ncomms11767. doi: 10.1038/ncomms11767 (PMC4909952; doi:10.1038/ncomms11767)
Supplement: Supplementary Information — Supplementary Figures 1-8, Supplementary Discussion and Supplementary References [file ncomms11767-s1.pdf]

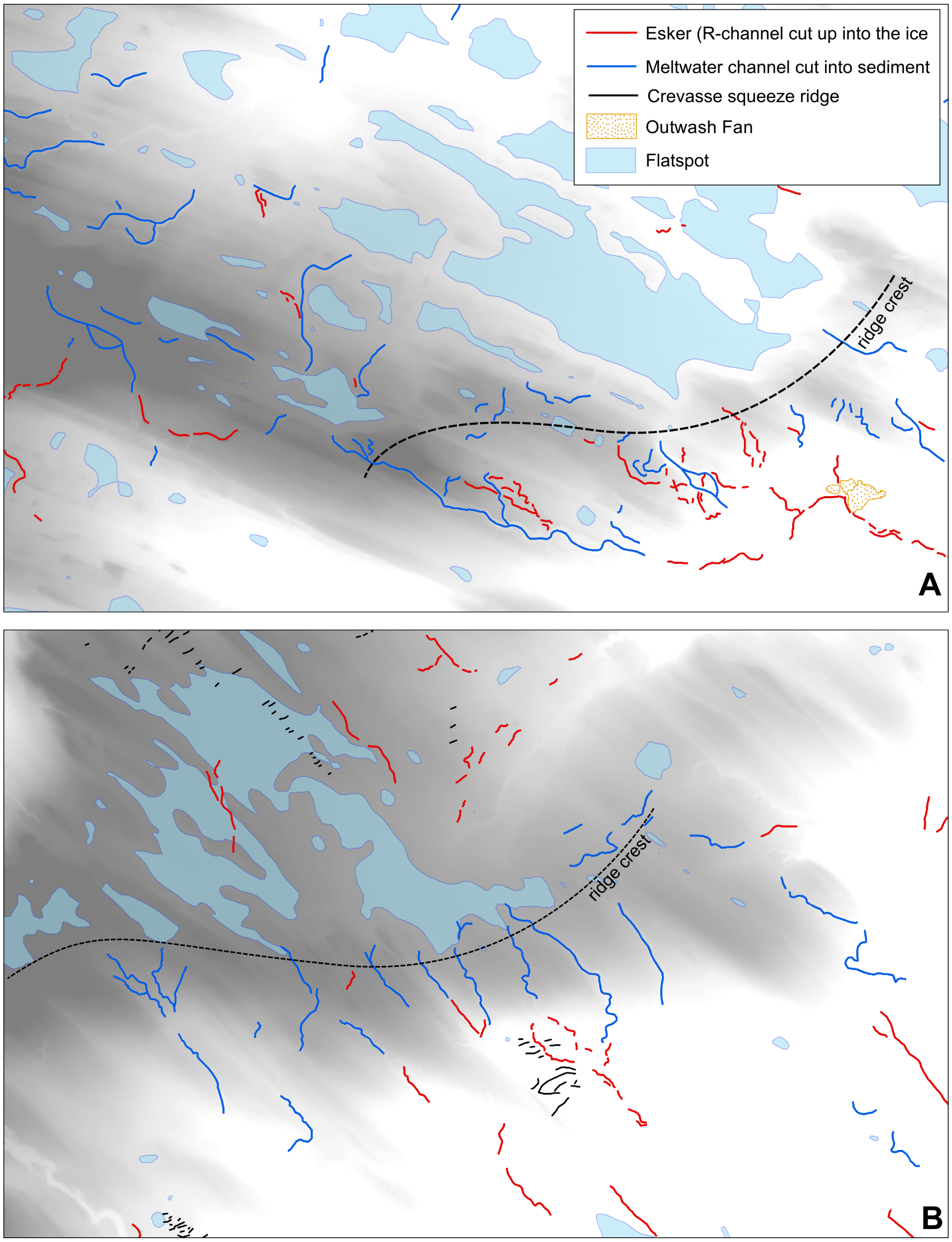

Supplementary Figure 1: **Mapped flat spots, meltwater channels, crevasse squeeze ridges, eskers and outwash fans.** **A**. Mapping of Site 1 (extent of Fig. 1B). **B**. Mapping of Site 2 (extent of Fig. 2A). The black dotted line is the ridge crest in both cases.

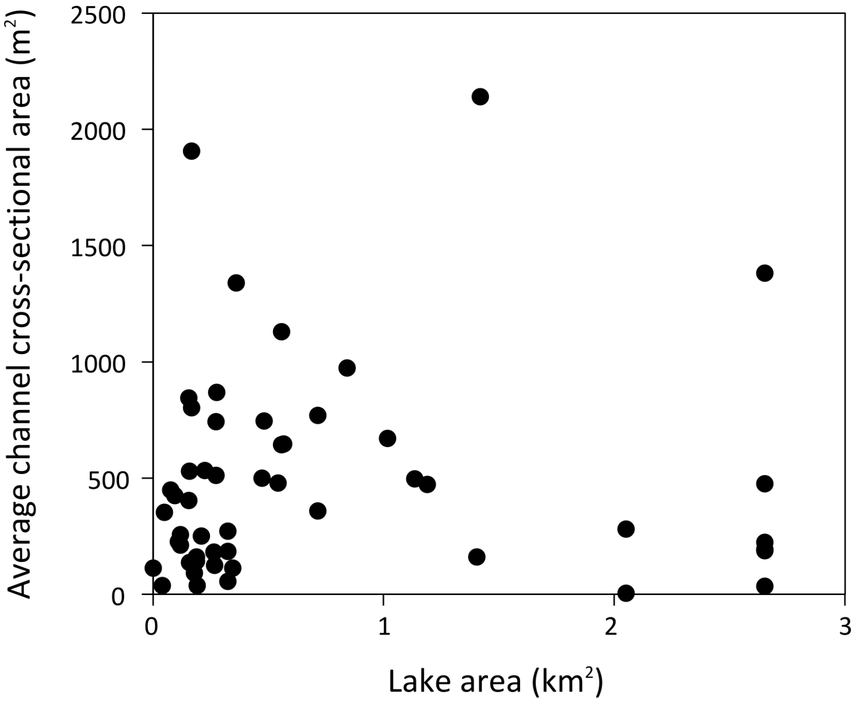

Supplementary Figure 2: Scatter plot showing the relationship between channel cross-sectional area (m^2) and lake area (km^2).

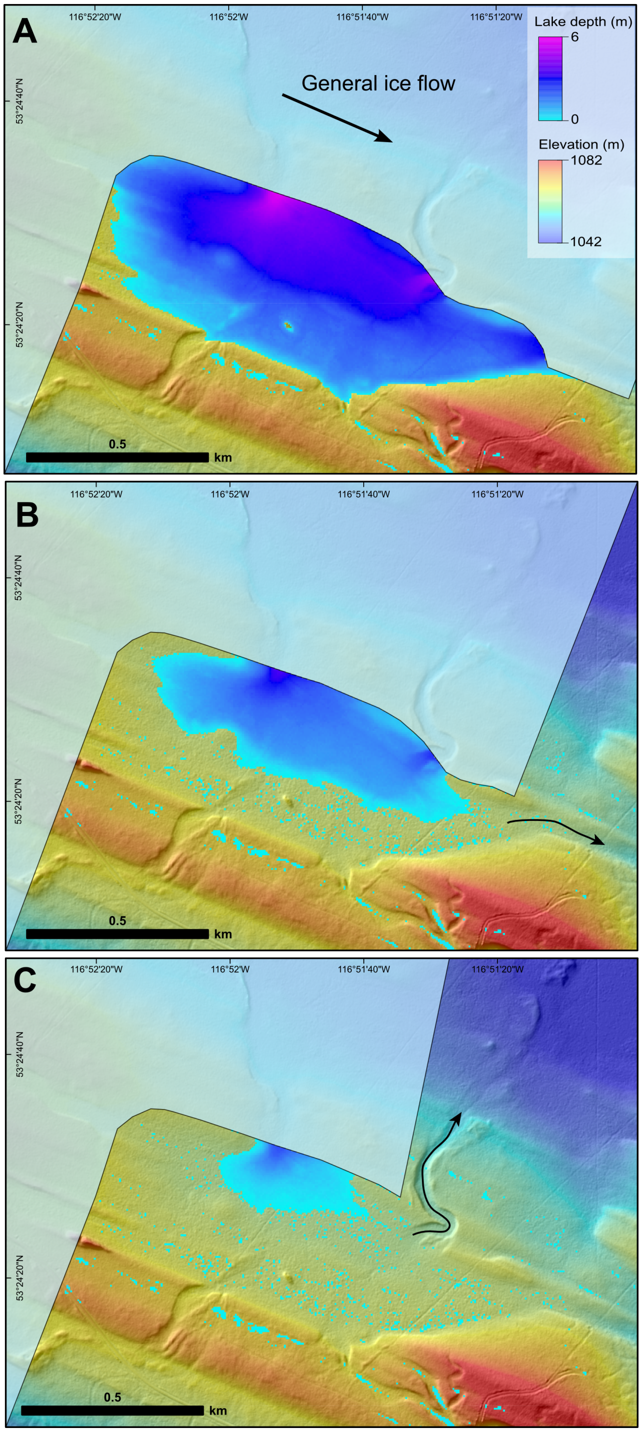

Supplementary Figure 3: **Scenarios for proglacial lake formation with different ice configurations (A-C).** It is only possible to pond a lake that covers the entire area of the flat spot if ice completely blocks the NE margin of the basin (**A**), otherwise water is able to drain out to the east (**B**) or north (**C**). It is difficult to envisage ice wrapping around the basin in this manner.

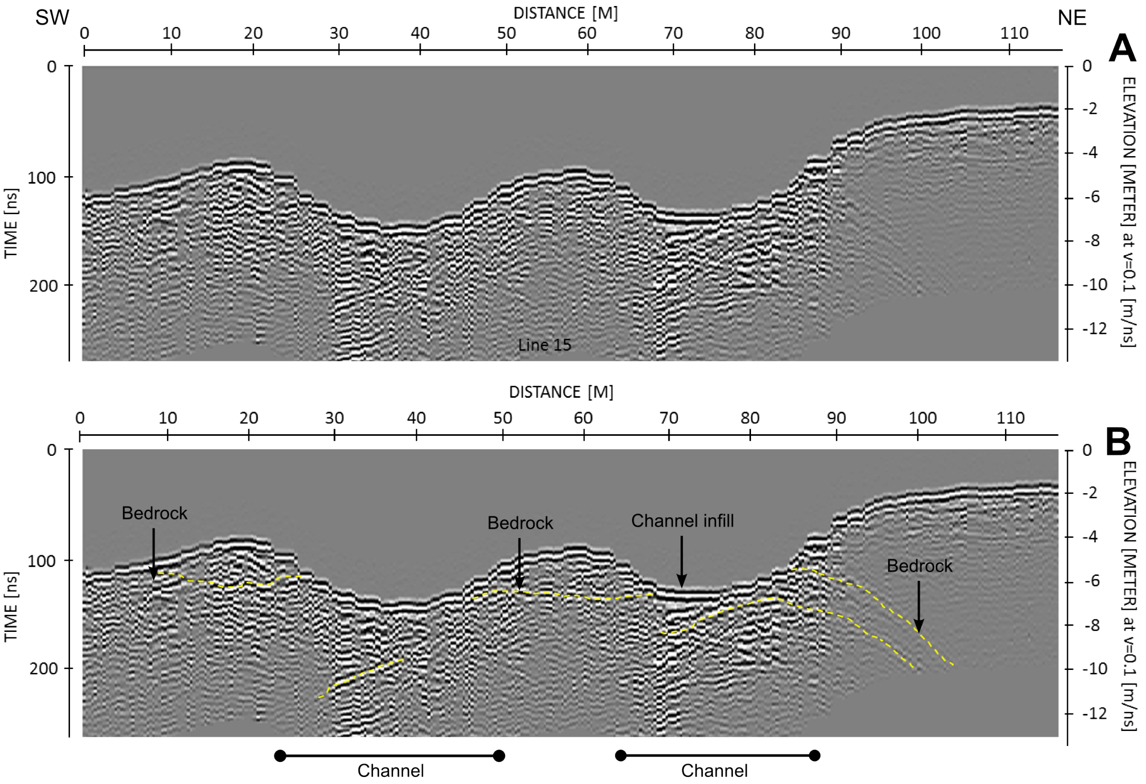

Supplementary Figure 4: **Ground penetrating radar profile 15 (raw in A, annotated in B) across subglacial meltwater channel at Site 1.** See **Figure 1B** for location of radar profile. Note the strong reflectors that outcrop roughly at the base of the channels. We interpret this to be bedrock. This suggests that the channel was cut primarily into sediment.

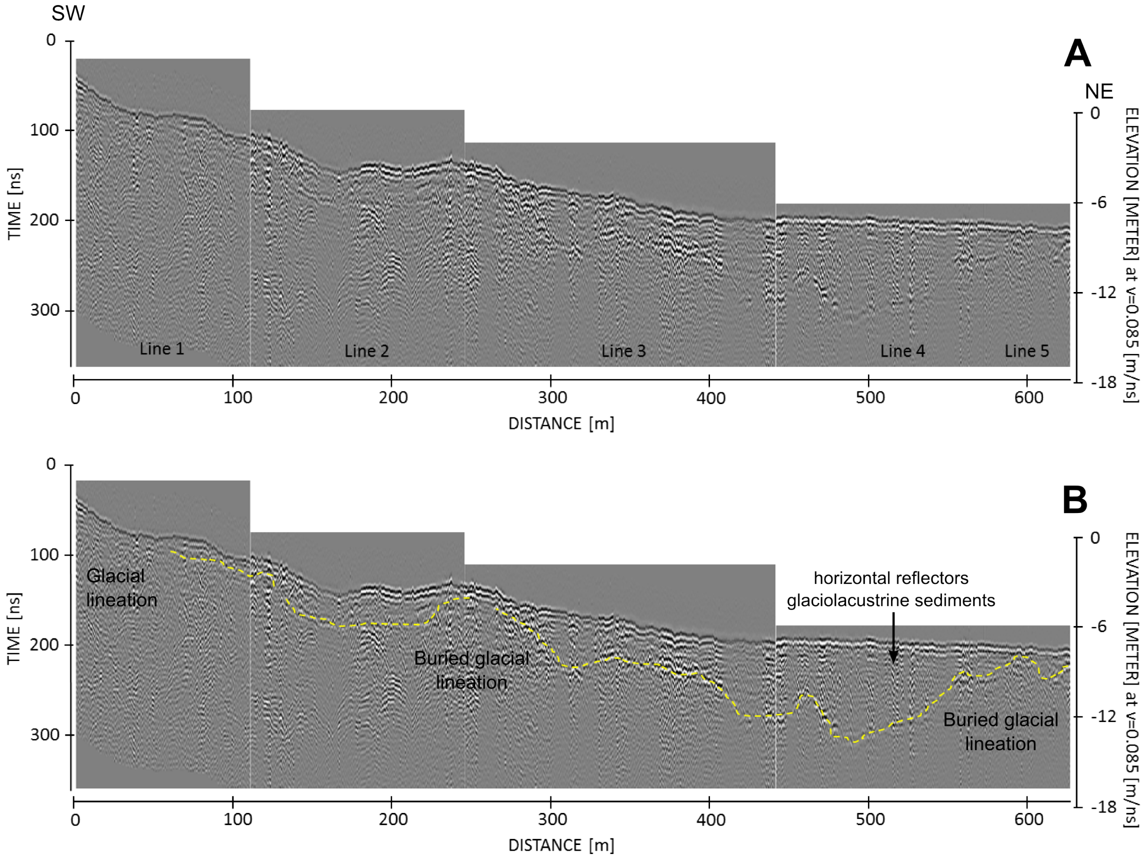

Supplementary Figure 5: **Ground penetrating radar profiles 1-5 (raw in A, annotated in B) across flat spot at Site 1.** See **Figure 1B** for location of radar profiles.

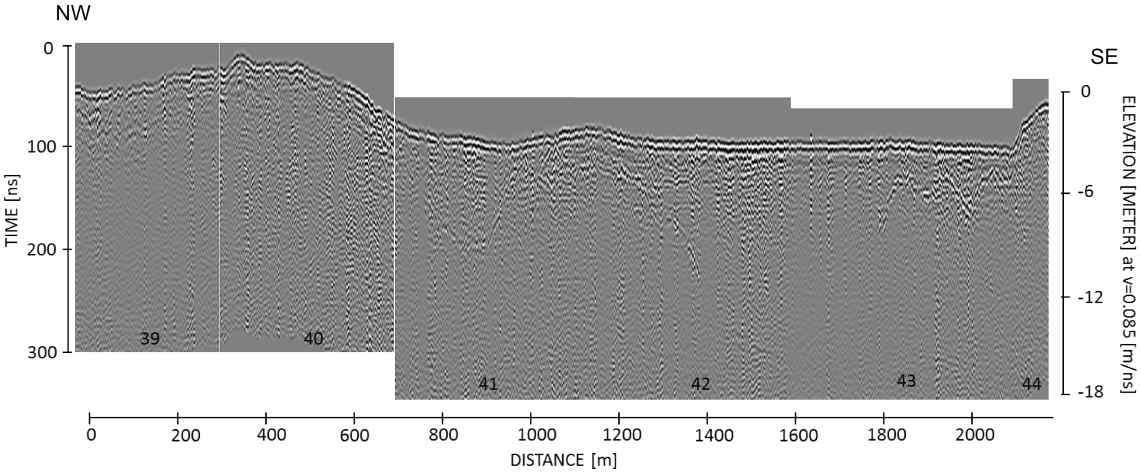

Supplementary Figure 6: **Raw ground penetrating radar profiles 39-44 across flat spot at Site 2.** See **Figure 2A** for location of radar profile and **Figure 2B** for interpretation.

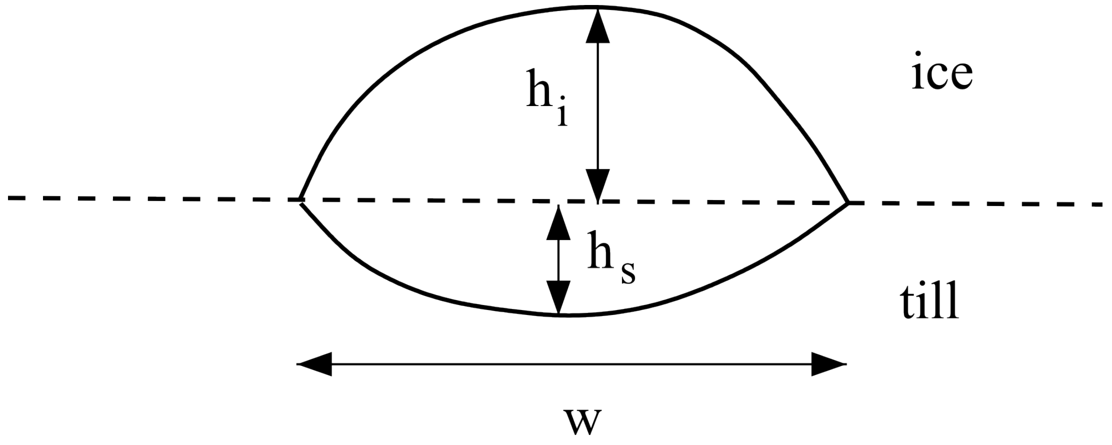

Supplementary Figure 7: **Generalised subglacial channel flow.** It consists of an incision of depth h_i upwards into the ice, and an incision of depth h_s downwards into the sediment; the latter is formed by erosion of the sediments, and the former by frictional melting of the ice.

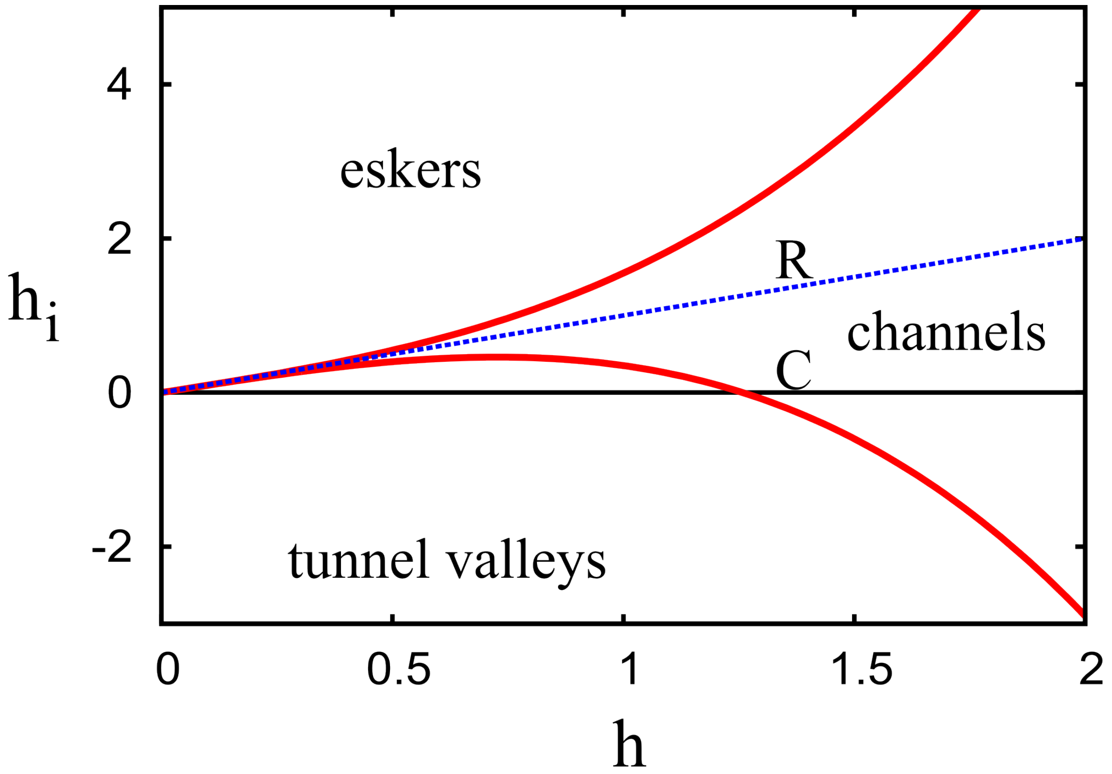

Supplementary Figure 8: **Ice roof elevation in metres as a function of channel depth in metres, based on** (6)**.** It uses values $V = 150$ m s^{-1}, $Q = 50$ m^3 s^{-1}, $r = 0.95$, and values of $\chi = 0.8$ (upper curve) and $\chi = 1.2$ (lower curve). Channels exist for small volume and sediment fluxes, but eskers or tunnel valleys develop at larger fluxes, depending on the stiffness of the till. The lines $h_\mathrm{i} = h$ (marked R) and $h_\mathrm{i} = 0$ (marked C) indicate pure Röthlisberger channels and canals, respectively.

Supplementary Discussion

The observations in Alberta are consistent with the idea that subglacial water flow ponds in lakes upstream of topographic ridges, and then drains through channelised flow for several kilometres downstream. It is consistent with our knowledge of other subglacial lakes that drainage occurs sporadically in subglacial floods[1–4], and we shall assume this is the case. This is likely to be the case deep under the ice sheet, as subglacial meltwater production is unlikely to be sufficient to fuel a permanent drainage channel. As the margin retreats, surface meltwater may enhance the basal water flow, but sporadic floods may still represent the normal drainage behaviour[5].

Efforts to provide a theoretical description of drainage below ice sheets have been made by, for example, Alley[6], Walder and Fowler[7], Ng[8,9] and Creyts and Schoof[10], but there has been little progress in describing the mechanisms whereby channelised flow may occur either as *canals* incised into the sediment, or as Röthlisberger (R) channels incised into the ice. An exception is in the thesis of Ng (ref. 11, chapter 7), whose detailed study provides an inspiration for the present discussion.The observations in the present paper appear to indicate that outflow from the lakes first occurs through canals, but then there is a downstream transition to R channels, manifested either through the disappearance of the canals, or in some cases by their mutation into eskers, which are taken as indicative of late stage infill of an R channel.

Without providing a complete theory, we show that these observations are consistent with what is presently theoretically known about subglacial floods, and also with the rudimentary theory of drainage given by Fowler (ref. 12, pp. 699 ff.), which we now summarise.

The geometry of the channels is shown in Supplementary Figure 7, and allows for the possibility of either an R channel ($h_{\rm s} = 0$, $h_{\rm i} > 0$) or a canal ($h_{\rm i} = 0$, $h_{\rm s} > 0$, or more generally a combination of both ($h_{\rm i} > 0$, $h_{\rm s} > 0$). In fact, there are further possibilities, as it is only necessary that the total channel depth

$$h = h_{\rm i} + h_{\rm s} \tag{1}$$

be positive; either $h_{\rm i}$ or $h_{\rm s}$ can be negative, and we associate the former possibility with the ice squeezing down into the sediment, and thus forming tunnel valleys in the way suggested by Boulton and Hindmarsh[13], and the latter (possibly) with the formation of eskers, as the sediment squeezes up into the ice.

Fowler (ref. 12, p. 701) generalises the classic closure equation of Röthlisberger[14] and Nye[15] to the two forms

$$\begin{aligned}\frac{\partial(wh_{\rm i})}{\partial t} &= \frac{\dot{m}_{\rm i}}{\rho_{\rm i}} - \frac{w^2}{\eta_{\rm i}}(N + \Delta\rho_{\rm wi} g h_{\rm i}),\\ \frac{\partial(wh_{\rm s})}{\partial t} &= \frac{\dot{m}_{\rm s}}{\rho_{\rm s}} - \frac{w^2}{\eta_{\rm s}}(N + \Delta\rho_{\rm sw} g h_{\rm s}),\end{aligned} \tag{2}$$

where N is the effective pressure at the ice/till interface far from the channel, and as shown in Supplementary Figure 7, w is the channel width, and $\dot{m}_{\rm i}$ and $\dot{m}_{\rm s}$ are the ice melt rate and the sediment erosion rate; $\rho_{\rm i}$ and $\rho_{\rm s}$ are ice and sediment densities, and

$\Delta\rho_{\rm wi} = \rho_{\rm w} - \rho_{\rm i}$ and $\Delta\rho_{sw} = \rho_{\rm s} - \rho_{\rm w}$ are their differences from the water density $\rho_{\rm w}$. The closure of both ice and sediment is represented by viscosities $\eta_{\rm i}$ and $\eta_{\rm s}$, although particularly that for sediment is likely an over-simplification of realistic granular flow rheologies[16]. Both $\eta_{\rm i}$ and $\eta_{\rm s}$ will realistically depend on N, but in opposite ways: As N increases, $\eta_{\rm i}$ decreases due to Glen's law, but $\eta_{\rm s}$ increases as the yield stress increases.

We assume the thermal erosion (melting) rate and sediment erosion rate are given by the relations

$$\dot{m}_{\rm i} = C_{\rm i} Q, \quad \dot{m}_{\rm s} = C_{\rm s} Q, \tag{3}$$

the first of which equates the latent heat used to melt the ice wall to the rate of potential energy release (stream power) of the flow, and the second is proposed by analogy. The quantities Q and $Q_{\rm s}$ are stream and sediment flow, and are related to channel geometry by

$$Q = \sqrt{C} w h^{3/2}, \quad Q_{\rm s} = K' w (h - h_{\rm c})^{3/2}, \tag{4}$$

which represent respectively a turbulent friction law and a Meyer-Peter and Müller type bedload transport law. The constants C, $C_{\rm s}$ and $C_{\rm i}$ are given by Fowler[12]; specifically, we take

$$C_{\rm i} = \frac{\rho_{\rm i} g S_{\rm i}}{2L}, \quad C_{\rm s} = \frac{\rho_{\rm i} g S_{\rm i}}{2L_{\rm E}}, \quad C = \frac{\rho_{\rm i} g S_{\rm i}}{2f\rho_{\rm w}}, \tag{5}$$

where $S_{\rm i}$ is the ice upper surface slope, f is a friction factor, L is latent heat of melting, and $L_{\rm E}$ is a comparably defined 'work of erosion'.

In conventional flood theory[15,17], Q is determined by the refilling conditions in the upstream lake, and in the present case $Q_{\rm s}$ would be also. As the present aim is not to provide such a flood theory, we simply assume that Q and $Q_{\rm s}$ are prescribed. The equations in (1), (2), (3) and (4) then provide seven equations for the seven unknowns w, h, $h_{\rm i}$, $h_{\rm s}$, $\dot{m}_{\rm i}$, $\dot{m}_{\rm s}$ and N, which can be simply solved in steady state conditions.

What is of interest is the dependence of $h_{\rm i}$ on h, which itself depends on streamflow Q, and also the dependence of N on h. After some algebra, we find

$$h_{\rm i} = rh + \frac{V(1-\chi)h^3}{Q}, \tag{6}$$

where

$$r = \frac{\Delta\rho_{\rm sw}}{\Delta\rho_{\rm si}}, \quad V = \frac{\rho_{\rm i} g S_{\rm i}^2 \eta_{\rm i}}{4f\rho_{\rm w}\Delta\rho_{\rm si} L}, \quad \chi = \frac{\rho_{\rm i} L \eta_{\rm s}}{\rho_{\rm s} L_{\rm E} \eta_{\rm i}}, \tag{7}$$

and N is given by

$$N = \Delta\rho_{\rm sw} g h \left[V(1+\delta\chi)\frac{h^2}{Q} - \delta r \right], \tag{8}$$

where also

$$\delta = \frac{1-r}{r} = \frac{\Delta\rho_{\rm wi}}{\Delta\rho_{\rm sw}}. \tag{9}$$

If we use values $S_\mathrm{i} = 10^{-3}$, $\eta_\mathrm{i} = 10^{14}$ Pa s, $f = 0.005$, $\rho_\mathrm{s} = 2 \times 10^3$ kg m^{-3}, then typical values of the parameters are

$$V \sim 140 \text{ m s}^{-1}, \quad r \sim 0.92, \quad \delta \sim 0.08. \tag{10}$$

The value of χ depends on the notional erosion work term L_E, which is not well constrained. We use the experimental results of Begin *et al.*[18] to provide an estimate. Their supply term B is the equivalent of $\dfrac{\dot{m}_\mathrm{s}}{\rho_\mathrm{s} w}$, and they estimate $B \sim 0.04$ cm min^{-1} $= 0.7 \times 10^{-5}$ m s^{-1}. This implies, for their values of $\rho_\mathrm{s} \approx 1.5 \times 10^3$ kg m^{-3} and $w \sim 0.25$ m that $\dot{m}_\mathrm{s} \sim 2.5 \times 10^{-3}$ kg m^{-1} s^{-1}. The estimate for L_E then comes via (3) and (5),

$$L_\mathrm{E} \sim \frac{\rho_\mathrm{i} g S_\mathrm{i} Q}{\dot{m}_\mathrm{s}} \sim 30 \text{ J kg}^{-1}. \tag{11}$$

(The factor 2 is omitted as the experiments were of a free surface flow.)

Typical estimates for $\eta_\mathrm{s} \sim 10^{10}$ Pa s (ref. 19, table 2) suggest that $\eta_\mathrm{s}/\eta_\mathrm{i} \sim 10^{-4}$ while corresponding estimates for sediment flux[20] suggest that $Q_\mathrm{s}/Q \sim 10^{-4}$. Indeed, since (4) implies that

$$h = \frac{h_\mathrm{c}}{1 - \left(\dfrac{Q_\mathrm{s}}{\kappa Q}\right)^{2/3}}, \tag{12}$$

where

$$\kappa = \frac{K'}{\sqrt{C}} = \frac{\rho_\mathrm{i} K S_\mathrm{i} \sqrt{f}}{2 \Delta \rho_\mathrm{sw}} \sim 2.6 \times 10^{-4} \tag{13}$$

(ref. 12, p. 702), we require $Q_\mathrm{s}/Q < \kappa$. All these estimates combine to suggest $\chi \sim O(1)$. Given the uncertainty, $O(1)$ values of χ are apparently feasible.

Supplementary Figure 8 indicates the way in which h_i varies with stream depth h. A given ratio Q_s/Q determines h via (12), and for this h, the channel form largely depends on χ (see equation (6)): for $\chi < 1$, R channels are formed, while for $\chi > 1$, canals are formed.

The most obvious variation of χ defined in (7) is due to the variation of η_i and η_s with N. As N increases, η_i decreases roughly as $1/N^2$ (according to Glen's law), while the sediment viscosity can only increase (and reach infinity at a finite value when the yield stress is no longer exceeded). This suggests that χ is a strongly increasing function of N, and thus that the form of the channel downstream of a lake will depend critically on the profile of the effective pressure in the channel. In particular, a downstream transition from canals to R channels is consistent with this description if N is high ($\chi > 1$) upstream, and low ($\chi < 1$) downstream.

To assess this, we consider the elements of subglacial flood theory. This is described by Fowler (ref. 12, pp. 742 ff.). The essence of the matter is that the hydraulic gradient in the stream can be written in the form

$$\frac{\tau}{R} = \Phi + \frac{\partial N}{\partial x}, \tag{14}$$

where (cf. equation (4.8) of ref. 12) τ is the shear stress in the stream flow and $R \approx h$ is the hydraulic radius, which here is essentially the depth; x is downstream distance and

$$\Phi \approx \rho_{\rm i} g S_{\rm i} \tag{15}$$

(ref. 12, equation (11.10)) is the basic hydraulic gradient due to the ice surface slope and the bed slope. During a flood τ increases and then decreases, so that at the end of the flood,

$$\frac{\partial N}{\partial x} \approx -\Phi, \tag{16}$$

and we will use this to describe the expected form of the channel shape. Note, however, that (16) is only applicable within a relatively short distance of the lake; beyond this $\dfrac{\partial N}{\partial x} \to 0$, and (14) simply implies $\tau \approx h\Phi$.

One of the conclusions of the flood theory (e. g., ref. 21), and essential in explaining their periodicity, is that although generally $\Phi > 0$ (because the ice surface slope drives the stream flow in the same direction as ice flow), Φ will be negative in the vicinity of the lake (indeed, this is why there is a lake). In the present case, we associate this region of negative Φ with the topographic ridge, but we then expect that $\Phi > 0$ downstream of the ridge, and there is a fairly short region adjacent to the lake where $\dfrac{\partial N}{\partial x} < 0$. The length of this region can be estimated as $X \sim N/\Phi$, and using (15) and (8) in the form

$$N \sim \Delta\rho_{\rm sw} g h^*, \quad h^* = \sqrt{\frac{Q}{V}} \sim 0.6 \text{ m}, \tag{17}$$

this give

$$X \sim \frac{h^*}{S_{\rm i}} \sim 0.6 \text{ km}, \tag{18}$$

which is roughly consistent with the observed length scale of the stream features. Over this length N and thus also χ decreases downstream, and this is consistent with the observation of upstream canals making a transition to R channels downstream. It is not necessary as χ can decrease downstream without making the transition through $\chi = 1$, but it is consistent.

Supplementary References

1. Björnsson, H. Hydrology of ice caps in volcanic regions. Societas Scientarium Islandica, University of Iceland, Reykjavik (1988).

2. Björnsson, H. Jökulhlaups in Iceland: prediction, characteristics and simulation. *Ann. Glaciol.* **16**, 95–106 (1992).

3. Wingham, D. J., Siegert, M. J., Shepherd, A. & Muir, A. S. Rapid discharge connects Antarctic subglacial lakes. *Nature* **440**, 1,033–1,037 (2006).

4. Carter, S. P., Fricker, H. A. & Siegfried, M. R. Evidence of rapid subglacial water piracy under Whillans Ice Stream, West Antarctica. *J. Glaciol.* **59** (218), 1,147–1,162 (2013).

5. Goodwin, I. D. The nature and origin of a jökulhlaup near Casey Station, Antarctica. *J. Glaciol.* **34**, 95–101 (1988).

6. Alley, R. B. Water-pressure coupling of sliding and bed deformation: I. Water system. *J. Glaciol.* **35**, 108–118 (1989).

7. Walder, J. S. & Fowler, A. Channelised subglacial drainage over a deformable bed. *J. Glaciol.* **40**, 146–152 (1994).

8. Ng, F. S. L. Canals under sediment-based ice sheets. *Ann. Glaciol.* **30**, 3–15 (2000).

9. Ng, F. S. L. Coupled ice–till deformation near subglacial channels and cavities.*J. Glaciol.* **46**, 580–598 (2000).

10. Creyts, T. T. & Schoof, C. G. Drainage through subglacial water sheets. *J. Geophys. Res.* **114**, F04008, doi:10.1029/2008JF001215 (2009).

11. Ng, F. S. L. Mathematical modelling of subglacial drainage and erosion. D. Phil. thesis, University of Oxford (1998).

12. Fowler, A. C. Mathematical geoscience. Springer-Verlag, London (2011).

13. Boulton, G. S. & Hindmarsh, R. C. A. Sediment deformation beneath glaciers: rheology and geological consequences. *J. Geophys. Res.* **92** (B9), 9,059–9,082 (1987).

14. Röthlisberger, H. Water pressure in intra- and subglacial channels. *J. Glaciol.* **11**, 177–203 (1972).

15. Nye, J. F. Water flow in glaciers: jökulhlaups, tunnels, and veins. *J. Glaciol.* **17**, 181–207 (1976).

16. Jop, P., Forterre, Y. & Pouliquen, O. A constitutive law for dense granular flows. *Nature* **441**, 727–730 (2006).

17. Fowler, A. C. Dynamics of subglacial floods. *Proc. R. Soc. A***465**, 1,809–1,828 (2009).

18. Begin, Z. B.,Meyer, D. F. & Schumm, S. A. Development of longitudinal profiles of alluvial channels in response to base-level lowering. *Earth Surf. Proc. Landf.* **6** (1), 49–68 (1981).

19. Porter, P. R. & Murray, T. Mechanical and hydraulic properties of till beneath Bakaninbreen, Svalbard. *J. Glaciol.* **47** (157), 167–175 (2001).

20. Collins D. N. Sediment concentration in melt waters as an indicator of erosion processes beneath an Alpine glacier. *J. Glaciol.* **23**, 247–257 (1979).

21. Fowler, A. C. Breaking the seal at Grímsvötn, Iceland. *J. Glaciol.* **45**, 506–516 (1999).
